# CaSun1, a SUN family protein, governs the pathogenicity of *Colletotrichum camelliae* by recruiting CaAtg8 to promote mitophagy

**DOI:** 10.1093/hr/uhaf121

**Published:** 2025-05-02

**Authors:** Shuai Meng, Shufen Chao, Meng Xiong, Longjun Cheng, Yu Sun, Li Wang, Ya Chen, Sadhna Jagernath Jane, Chaoxi Luo, Jie Chen

**Affiliations:** National Joint Local Engineering Laboratory for High-Efficient Preparation of Biopesticide, College of Forestry and Biotechnology, Zhejiang Agriculture and Forestry University, No. 666 Wusu Street, Lin'an District, Hangzhou 311300, China; National Joint Local Engineering Laboratory for High-Efficient Preparation of Biopesticide, College of Forestry and Biotechnology, Zhejiang Agriculture and Forestry University, No. 666 Wusu Street, Lin'an District, Hangzhou 311300, China; Hubei Key Laboratory of Plant Pathology, and College of Plant Science and Technology, Huazhong Agricultural University, Shizishan Street No.1, Wuhan 430070, China; National Joint Local Engineering Laboratory for High-Efficient Preparation of Biopesticide, College of Forestry and Biotechnology, Zhejiang Agriculture and Forestry University, No. 666 Wusu Street, Lin'an District, Hangzhou 311300, China; National Joint Local Engineering Laboratory for High-Efficient Preparation of Biopesticide, College of Forestry and Biotechnology, Zhejiang Agriculture and Forestry University, No. 666 Wusu Street, Lin'an District, Hangzhou 311300, China; Zhengzhou Fruit Research Institute, Chinese Academy of Agricultural Sciences, Zhengzhou, Henan 450009, China; State Key Laboratory of Rice Biology, China National Rice Research Institute, 28 Dao Ru Rice, Fuyang District, Hangzhou 311400, China; Agricultural Department, Faculty of Technological Sciences, Anton de Kom University of Suriname, Leysweg 86, Building 7, Paramaribo, Suriname; Hubei Key Laboratory of Plant Pathology, and College of Plant Science and Technology, Huazhong Agricultural University, Shizishan Street No.1, Wuhan 430070, China; National Joint Local Engineering Laboratory for High-Efficient Preparation of Biopesticide, College of Forestry and Biotechnology, Zhejiang Agriculture and Forestry University, No. 666 Wusu Street, Lin'an District, Hangzhou 311300, China

## Abstract

*Camellia oleifera*, a woody oilseed plant native to China, is highly susceptible to anthracnose, a fungal disease that poses a significant threat to its yield and quality. Mitophagy, a specialized form of autophagy that specifically targets dysfunctional mitochondria, is crucial for cellular homeostasis, stress response, and pathogenesis in fungi. The proteins that potentially participate in mitophagy in *Colletotrichum camelliae* were identified herein using immunoprecipitation-mass spectrometry (IP-MS) by screening for the potential protein interactors of the core autophagy-related protein, CaAtg8. Among the identified mitochondria-associated proteins, CaSun1 was selected for further investigation. Phenotypic analyses revealed that CaSun1 is a critical regulator of vegetative growth, conidiation, and pathogenicity. CaSun1 localized to the mitochondria, consistent with the conserved function of SUN family proteins. Notably, the findings revealed that CaSun1 was essential for mitophagy and colocalized with CaAtg8 during nitrogen starvation. Functional analyses demonstrated that CaSun1-mediated mitophagy is vital for the growth of invasive hyphae and pathogenicity in *C. camelliae*. In summary, our findings indicated that CaSun1 mediates mitophagy by facilitating the recruitment of CaAtg8 in *C. camelliae*, thereby contributing to the establishment of anthracnose. This study provided novel insights into the molecular mechanisms underlying the pathogenesis of fungal infections and identified a potential target for disease control.

## Introduction

Mitophagy is a selective form of autophagy in which dysfunctional or superfluous mitochondria are specifically targeted for degradation [1]. This process is crucial for maintaining mitochondrial health, regulating energy production, and preventing the accumulation of harmful reactive oxygen species (ROS) [2, 3]. Similar to that of other eukaryotes, fungal mitophagy plays a crucial role in cellular adaptation to environmental changes, stress responses, and microbial infection [4, 5]. Recent studies have highlighted the essential role of mitophagy in the vegetative growth and pathogenicity of plant-pathogenic fungi [6, 7]. MoAtg24 and MoWhi2, the mitophagy-related proteins of *Magnaporthe oryzae*, regulate mitophagy in the foot cells and invasive hyphae, thereby influencing conidiation and virulence [7, 8]. The mitochondrial fission/fusion-related proteins of *M. oryzae*, including MoDnm1, MoFis1, MoMdv1, and MoFzo1, also contribute to mitophagy and virulence [6, 9]. The *MoAUH1* gene, which encodes 3-methylglutaconyl-CoA hydratase, facilitates mitophagy to regulate the virulence of *M. oryzae* [10]. It has been recently identified that the MoAtg8-interacting protein MoAti1 is essential for mitophagy during the development of invasive hyphae, affecting the pathogenicity of *M. oryzae* [11]. AoAtg26, the putative sterol glucosyltransferase of *Aspergillus oryzae*, critically regulates mitophagy, vegetative development, and conidiation [12]. Similarly, UvSnx4, a SNX family protein of *Ustilaginoidea virens*, the primary pathogen responsible for rice false smut, regulates mitophagy in the secondary spores, stress responses, and virulence [13].

Atg8, referred to as LC3 (microtubule-associated protein 1A/1B-light chain 3), plays a crucial role in the autophagic processes of eukaryotic cells [14]. It interacts with various proteins through specific motifs known as Atg8-interacting motifs (AIMs) or LC3-interacting regions (LIRs) [15, 16]. These motifs are essential for the recognition and binding of Atg8/LC3, which facilitate the recruitment of proteins to autophagosomes and promote autophagy [11]. Mitophagy involves the selective degradation of mitochondria by autophagy, in which superfluous or dysfunctional mitochondria are engulfed by autophagosomes for depolarization and subsequent elimination. Mitophagy is therefore essential for conserving vital nutrients and preventing their excessive utilization, which consequently mitigates the generation of ROS. This process is necessary for maintaining intracellular homeostasis, promoting cell survival, and supporting proper growth and differentiation [2, 3]. Despite advancements in understanding the biological functions of mitophagy in yeast, the molecular mechanisms underlying mitophagy in pathogenic fungi remain poorly defined.

Anthracnose, caused by *Colletotrichum camelliae*, significantly affects the yield and quality of tea oil tree [17, 18], making it essential to investigate the mechanism of pathogenesis of *C. camelliae*. During infection, the two-celled conidia of *C. camelliae* land on the surface of *Camellia oleifera* leaves, where they rapidly germinate and adapt to the environment. The conidia subsequently develop specialized structures called appressoria, which generate sufficient turgor pressure to penetrate the cuticles of the host plant [19–21]. Once inside the host plant, *C. camelliae* proliferates extensively by secreting various enzymatic components and secondary metabolites that degrade plant cell walls and disrupt the cellular metabolic processes of the host [22]. *Colletotrichum camelliae* subsequently differentiates into invasive hyphae that spread to adjacent cells, leading to the formation of lesion symptom [23, 24]. In *Colletotrichum fructicola*, CfGcn5 functions as an autophagy repressor, regulates autophagy during the formation of appressoria, and the generation of turgor pressure, which are essential for pathogenesis [25]. Additionally, the cysteine protease CfAtg4 interacts with CaAtg8 to regulate autophagy, thereby influencing growth and pathogenicity [26]. In *Colletotrichum higginsianum*, ChPhb1 and ChPhb2 interact with ChAtg24 to facilitate mitophagy in invasive hyphae during pathogenesis [27]. It has been additionally demonstrated that CaEch1-mediated mitophagy regulates vegetative growth, conidiation, appressorium formation, and pathogenicity in *C. camelliae*, highlighting the significance of mitophagy in the growth of invasive hyphae [28]. However, the molecular mechanisms underlying the pathogenesis of mitophagy in *C. camelliae* remain unknown.

The Sim1, Uth1, Nca3, and Sun4 proteins, members of the SUN protein family in yeast, exhibit considerable sequence similarity in their extended C-terminal domains, which comprise 258 amino acids [11, 29]. These proteins are implicated in various cellular processes, including mitochondrial morphology, stress response, and cell senescence [30]. Specifically, the deletion of the *UTH1* gene in yeast has been shown to inhibit the degradation of mitochondrial proteins induced by starvation or rapamycin (TOR), suggesting that Uth1 plays a crucial role in the selective removal of mitochondria [31]; however, the precise underlying mechanism remains to be elucidated. CaSun1, a member of the SUN protein family, was identified in this study using immunoprecipitation-mass spectrometry (IP-MS), with a *GFP-CaATG8* strain in *C. camelliae*. The findings revealed that CaSun1 localizes to the mitochondria and regulates asexual development, conidiation, stress responses, and virulence. We further confirmed that CaSun1 interacts with CaAtg8 via 1,3 AIM/LIR motifs and specifically regulates mitophagy by recruiting CaAtg8 to the mitochondria under nitrogen starvation. The findings suggest that CaSun1-mediated mitophagy is essential for virulence, emphasizing its significance in this important anthracnose-causing pathogen.

## Results

### CaSun1 is essential for vegetative growth, conidiation, and virulence

**Figure 1:**
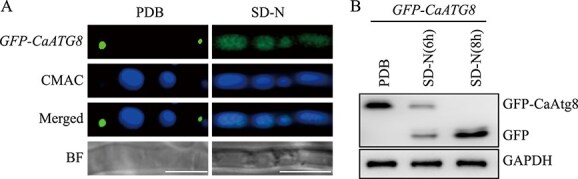
**Monitoring of autophagy using GFP-CaAtg8 by confocal microscopy and western blot.** (**A**) Confocal microscopy image of the *GFP-CaATG8* strain under nitrogen-rich and starvation condition. The *GFP-CaATG8* strain was inoculated in the PDB medium for 40 h and transferred into SD-N medium for 12 h. The vacuoles were stained with CMAC (7-amino-4-choromethylcoumarin). Scale bar, 5 μm. (**B**) The degradation of the GFP-CaAtg8 was observed under nitrogen starvation condition by western blot with an anti-GFP antibody. GAPDH was used as an internal reference.

Autophagy is a fundamental and evolutionarily conserved mechanism of cellular degradation that plays a crucial role in fungal virulence. Atg8, the only homolog of mammalian LC3 or yeast Atg8 in *C. camelliae*, functions as a core autophagy-related protein that is essential for selective autophagy. In this study, the *GFP-CaATG8* strain was constructed and confirmed by observing the cleavage of GFP-CaAtg8 under conditions of nitrogen starvation (Fig. 1). Mitophagy is a specialized form of autophagy in which dysfunctional mitochondria are targeted for degradation, and this process is tightly regulated by various signaling pathways. The potential proteins involved in mitophagy and their regulatory effects on the cellular processes of *C. camelliae* were investigated by IP-MS for identifying the potential protein interactors of CaAtg8 in the *GFP-CaATG8* strain. Approximately 32 potential protein interactors of CaAtg8 were identified in the immunoprecipitate, which exhibited high Sequest HT scores (Table S2). The beta-glucosidase protein had the highest number of unique peptides and the highest Sequest HT score, and exhibited significant homology to the mitochondrial protein, Uth1, identified from yeast [31]. Sequence comparison revealed that one candidate protein, CaSun1, shared sequence similarities with the SUN domain protein of *Saccharomyces cerevisiae* (Fig. S1A and B).

**Figure 2:**
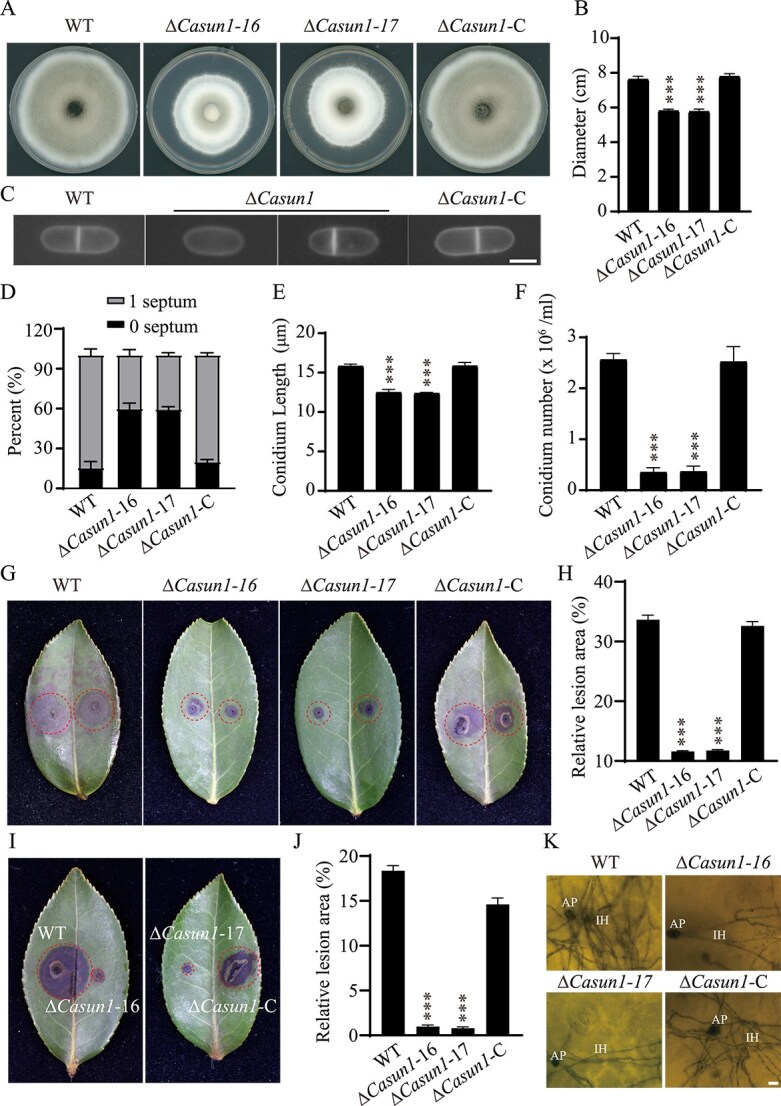
**CaSun1 is involved in fungal growth, conidiation, and virulence.** (**A**) The colony morphologies of WT, Δ*Casun1*, and complemented strains were cultured on PDA at 25°C for 7 days, and then photographed. (**B**) The colony diameters of the indicated strains. (**C**) The conidia morphology of WT, Δ*Casun1*, and complemented strain were measured. Bar: 5 μm. (**D**) The septum of the WT, Δ*Casun1*, and complemented strains were counted. (**E**) The conidia length of the indicated strains. (**F**) The conidium number of each strain were counted. (**G, I**) Disease symptoms on leaves of *C. oleifera* inoculated with conidial suspensions from WT, Δ*Casun1*, and Δ*Casun1*-C strains in a 25°C humidified chamber with a 14-h light and 10-h dark cycle. (**H, J**) After 7 days, quantification analysis of the lesion area on inoculated leaves were performed, with data showing mean ± SD from three independent experiments, each involving at least 20 leaves (*P* < 0.001). (**K**) Microscopic examination of invasive hyphae with aniline blue. AP, appressorium, IH, invasive hyphae. Scale bar, 10 μm.

To elucidate the physiological functions of CaSun1, the *CaSUN1* gene was deleted using a homologous recombination strategy combined with the ATMT method. The *CaSUN1* gene was replaced by a *hygromycin resistant cassette* (*HYG*) gene of *C. camelliae* (Fig. S2A). Three deletion mutants were confirmed by Southern blotting and qRT-PCR (Fig. S2B–D). Two mutant strains, Δ*Casun1*–16 and Δ*Casun1*–17, and a Δ*Casun1*-C complementation strain were selected for further analysis. The colony diameters of the Δ*Casun1* strains were smaller and they exhibited fewer conidia compared to those of the WT strain cultured on PDA medium at 25°C; however, these defects were restored in the Δ*Casun1*-C complementation strain (Fig. 2A, B, and F). Interestingly, the conidia of the Δ*Casun1* strains were short and exhibited abnormalities compared to those of the WT and Δ*Casun1*-C strains (Fig. 2C–E). Altogether, the findings suggest that CaSun1 plays a key role in fungal growth and conidial morphology.

The expression of *CaSUN1* was upregulated during infection with *C. camelliae*, implying that CaSun1 plays a key role in pathogenesis (Fig. S1C). Leaves from the susceptible Changlin 40 cultivar of *C. oleifera* were further subjected to virulence assays for determining whether CaSun1 regulates the pathogenicity of *C. camelliae*. The leaves of *C. oleifera* were inoculated with conidial suspensions of the Δ*Casun1*, WT, and complementation strains. The lesions caused by infection with the Δ*Casun1* mutant strain were smaller than those of the WT and complementation strains at 7 days post-inoculation (dpi) (Fig. 2G–J). Microscopic observations revealed that the invasive hyphae of the Δ*Casun1* strains were obviously hindered compared to those of the WT and Δ*Casun1*-C strains (Fig. 2K). Altogether, these data indicate that CaSun1 plays an essential role in the virulence of *C. camelliae*.

### CaAtg8 interacts with CaSun1

**Figure 3:**
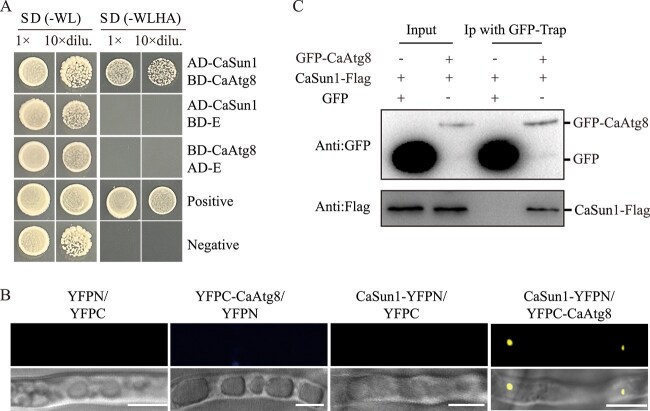
**CaAtg8 interacts with CaSun1.** (**A**) The interaction between CaAtg8 and CaSun1 was tested by yeast two-hybrid assays. The AD and BD vectors were cotransformed into AH109 Gold yeast cells, cultured on SD-Leu-Trp medium for 3 days, then diluted and dropped on SD-Trp/Leu and SD-Trp/Leu/Ade/His plates, respectively. (**B**) BiFC assay for examining the interaction between CaAtg8 and CaSun1. Yellow fluorescent protein (YFP) was split into YN and YC, then fused with CaSun1 or CaAtg8 to create CaSun1-YN and YC-CaAtg8. Fluorescence was observed only when CaSun1-YN was coexpressed with YC-CaAtg8. Scale bar, 5 μm. (**C**) Co-IP assay for confirming the interaction between CaAtg8 and CaSun1. The fusion protein CaSun1-Flag was detected in the eluted solution of the coincubated mixture of GFP-CaAtg8/CaSun1-Flag or GFP.

The interaction between CaAtg8 with CaSun1 was validated by yeast two-hybrid assays, which revealed a direct interaction between the proteins (Fig. 3A). The BiFC assays further confirmed a strong interaction between CaAtg8 and CaSun1 (Fig. 3B). The interaction was further validated by Co-IP assays, in which the GFP-CaAtg8/CaSun1-Flag or GFP/CaSun1-Flag proteins were separately inoculated with GFP-beads. The CaSun1 protein bands were subsequently detected following lysis of the GFP-CaAtg8 or GFP immunoprecipitates (Fig. 3C). These results demonstrated that CaAtg8 interacts with CaSun1 both *in vitro* and *in vivo*.

### CaAtg8 participates in conidiation, appressoria formation, and pathogenesis

Previous studies have demonstrated that Atg8 exhibits conserved biological functions in fungi [26, 35]. A phylogenetic tree was therefore constructed in this study by BLASTp search of the Atg8 homologs in other fungi, with the Atg8 protein of *S. cerevisiae* as the query, indicating that Atg8 is highly conserved across different fungi (Fig. S3A and B). It was observed that the expression level of *CaATG8* was markedly upregulated during infection with *C. camelliae*, suggesting its potential role in pathogenesis (Fig. S3C). The *CaATG8* gene was knocked out in the WT strain to explore the biological functions of CaAtg8. The phenotypes of both the deletion mutants were found to be comparable, and one mutant was selected for further analyses of the biological functions. Analysis of mycelial growth revealed that the colony diameter of the Δ*Caatg8* strains was comparable to that of the WT strain (Fig. S3D and E). However, the production of conidial decreased drastically in the Δ*Caatg8* mutant, compared to that of the WT strain after 7 days of growth in PDA plates (Fig. S3F). Subsequent assessment of appressoria formation and virulence revealed that the formation of appressoria were delayed in the Δ*Caatg8* mutant strains (Fig. S2G) and the areas of the lesions on the inoculated leaves were smaller compared to those caused by infection with the WT, Δ*Caatg8*-C, or *GFP-CaATG8* strains (Fig. S3H). These findings suggested that CaAtg8 is crucial for conidiation, appressoria development, and virulence in *C. camelliae*.

### CaSun1 participates in the stress response

Fungi must typically withstand various stresses, including temperature fluctuations, pesticides, and fertilizers, to achieve normal growth and infection [10, 36]. Therefore, the physiological function of a gene can be critically determined by assessing whether it plays a role in the stress response. Atg8 is known to aid in fungal adaptation to hostile environments and facilitate the utilization of cellular components during infections, and the present study verified that CaAtg8 interacts with CaSun1. To assess the role of CaSun1 in the response to various stresses, the WT, Δ*Casun1*, and Δ*Casun1*-C strains were cultured on PDA plates supplemented with a salt stressor (0.4 M NaCl) or cell wall stressors (0.04% SDS, 200 μg/ml CR, and 200 μg/ml CFW). Following a 7-day incubation period at 25°C, the Δ*Casun1* mutant displayed decreased tolerance to different cell wall stressors, including SDS, CR, and CFW. In addition, the growth of the WT and Δ*Casun1* mutant strains reduced by 8% and 12%, respectively, in the presence of 0.4 M NaCl (Fig. S4). The stress response phenotypes of the complementation strain were similar to those of the WT strain. These findings revealed that CaSun1 functions as a major regulator of the stress response in *C. camelliae*.

### CaSun1 is not essential for autophagy

**Figure 4:**
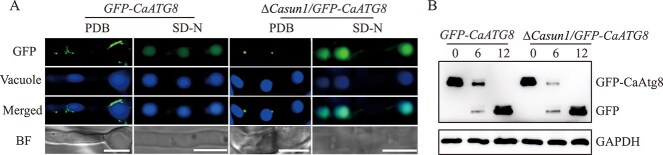
**CaSun1 is not required for autophagy under nitrogen starvation.** (**A**) Autophagy occurs in the Δ*Casun1* mutant. Mycelia of *GFP-CaATG8* and Δ*Casun1*/*GFP-CaATG8* strains were cultured in PDB for 40 h, and then shifted to SD-N medium for 6 or 12 h. The vacuoles were stained with CMAC (7-amino-4-choromethylcoumarin) prior to observation under confocal microscopy. Scale bar, 5 μm. (**B**) The degradation of GFP-CaAtg8 fusion protein was assessed by immunoblotting with an anti-GFP antibody, with GAPDH serving as an internal control.

The Atg8 protein, which is homologous to the mammalian LC3 protein, is crucial for autophagy in fungi. However, the present study revealed that CaSun1 interacts with CaAtg8, prompting further investigation into whether CaSun1 plays a regulatory role in autophagy. The GFP-CaAtg8 fusion protein, a marker for autophagy, was expressed in the WT and Δ*Casun1* strains, and the autophagic flux was subsequently assessed by fluorescence microscopy and western blotting. The findings revealed that when grown in a nutrient-rich medium, the fluorescence due to *GFP-CaATG8* and Δ*Casun1*/*GFP-CaATG8* was predominantly localized in the cytoplasm, and puncta were observed around the vacuoles, which were stained with 7-amino-4-chloromenthylcoumarin (CMAC) dye. However, following the transfer of hyphae from *GFP-CaATG8* and Δ*Casun1*/*GFP-CaATG8* to a nitrogen-starved SD-N medium for 6 h, the fluorescence due to *GFP-CaATG8* and Δ*Casun1*/*GFP-CaATG8* was predominantly observed within the vacuoles (Fig. 4A). The autophagic flux was subsequently determined via western blotting by measuring the expression of free GFP and GFP-CaAtg8 fusion proteins. The results demonstrated that the degradation of Δ*Casun1*/*GFP-CaATG8* was comparable to that of *GFP-CaATG8* (Fig. 4B), indicating that CaSun1 is not essential for autophagy.

### CaSun1 localizes to mitochondria and participates in mitophagy

**Figure 5:**
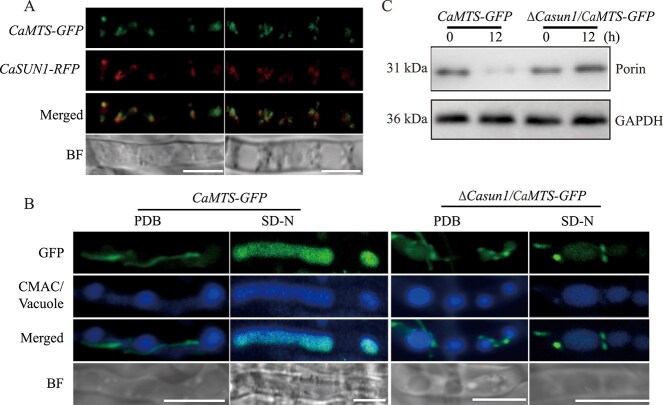
**CaSun1 localizes to mitochondria and is involved in mitophagy.** (**A**) *CaSUN1-RFP* colocalized with mitochondria marker *CaMTS-GFP* on the punctate and tubular structures in the hyphae. Scale bar, 5 μm. (**B**) Mitophagy occurs in Δ*Casun1* strain. Mycelia cultured in PDB for 40 h were subjected to SD-N medium for 12 h. The mycelia were stained with CMAC before confocal microscopic observation. (**C**) Immunoblot analysis to detect the degradation of mitochondria. Equal amounts of mycelia of each strain were performed immunoblot with anti-Porin antibody. GAPDH was used as a loading control.

The subcellular localization of CaSun1 was subsequently investigated in this study. To this end, the results of previous research were considered, which revealed that the proteins in the SUN domain family localize to the mitochondria in yeast [30, 31]. Subsequent prediction with the PSORT II webserver revealed that CaSun1 predominantly localizes in the mitochondria. The *CaSUN1-RFP* fusion protein was coexpressed with *CaMTS-GFP*, a marker of mitophagy, in the WT strain. The strong colocalization of the green (*CaMTS-GFP*) and red (*CaSUN1-RFP*) fluorescence signals suggested that CaSun1 localized within the mitochondria in *C. camelliae* (Fig. 5A).

Given that CaSun1 localizes within mitochondria and interacts with CaAtg8, we sought to investigate whether CaSun1 plays a regulatory role in mitophagy. To this end, the *CaSUN1* gene was deleted in the *CaMTS-GFP* strain [37, 38]. The fluorescence due to *CaMTS-GFP* appeared more predominant in the tubular mitochondria surrounding the vacuoles in the WT and Δ*Casun1* strains following culture in the nutrient-rich medium. After 6 h of nitrogen starvation, the fluorescence due to GFP was predominantly observed in the vacuoles of the WT strain; however, it was rarely detected in the vacuoles of the Δ*Casun1* mutant strains, indicating that CaSun1 plays an essential role in mitophagy (Fig. 5B). The degradation of CaMts-GFP was further evaluated by western blotting by assessing the total amount of porin, a mitochondrial marker protein [8, 9]. The porin bands were predominant in the CaMts-GFP and Δ*Casun1*/*CaMTS-GFP* strains under conditions of nitrogen starvation; however, the bands appeared more stable in the Δ*Casun1*/*CaMTS-GFP* strain than in the *CaMTS-GFP* strain (Fig. 5C). Altogether, these findings suggested that CaSun1 plays an essential role in mitophagy.

### CaSun1 recruits CaAtg8 to mitochondria by binding to AIM/LIR motifs

**Figure 6:**
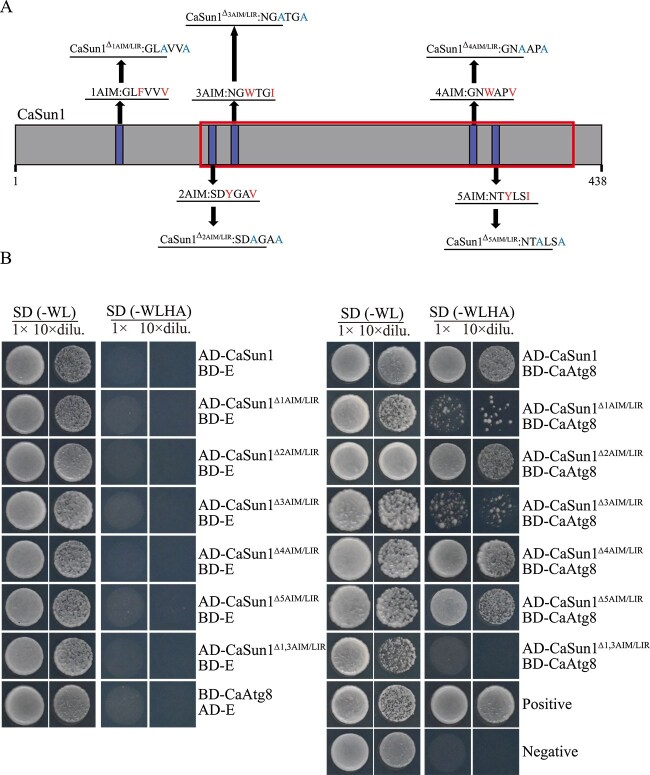
**CaSun1 interacts with CaAtg8 via the first and third AIM/LIR motifs.** (**A**) The AIM/LIR motifs of CaSun1 with the SUN domain highlighted in a red box. (**B**) Yeast two-hybrid assays were performed to assess the interaction between CaSun1 and CaAtg8. Mutations in the AIM/LIR motifs, where conserved amino acids (in red) were replaced with alanine amino acids (in blue), are shown in (A). The first and third motif mutations resulted in weaker interactions between CaSun1 and CaAtg8 than WT CaSun1.

It has been reported that several proteins interact with Atg8/LC3 via AIM/LIR motifs [15]. To determine whether CaSun1 contains any potential AIM/LIR motifs, its protein sequence was analyzed using the iLIR database, which revealed five potential AIM/LIR motifs in CaSun1 (Fig. 6A). Of these, the motifs that mediate binding to CaAtg8 were further identified by generating CaSun1 point mutants, which were subsequently cloned into the pGADT7 vector. The results of yeast two-hybrid assays revealed that mutations in the first and third AIM/LIR motifs weakened the interaction between CaSun1 and CaAtg8 (Fig. 6B). It was therefore inferred that these two AIM/LIR motifs of CaSun1 are critical for its binding to CaAtg8. Furthermore, the *CaSUN1*^Δ*1,3AIM*/*LIR*^*-RFP* fusion protein, which was coexpressed in the *GFP-CaATG8* strain, colocalized with *GFP-CaATG8* and *CaSUN1-RFP*. However, the green and red fluorescent signals of *GFP-CaATG8* and *CaSUN1*^Δ*1,3AIM*/*LIR*^*-RFP*, respectively, failed to overlap under conditions of nitrogen starvation (Fig. S5A), which was consistent with the results of yeast two-hybrid analysis, indicating that the first and third AIM/LIR motifs of CaSun1 are essential for its interaction with CaAtg8.

The functions of those two AIM/LIR motifs were further investigated by generating point mutants of CaSun1 in the WT strain for analyzing the phenotypic changes. The growth of the *CaSUN1*^Δ*1,3AIM*/*LIR*^*-C*/Δ*Casun1* mutants was reduced and conidiation reduced markedly compared to those of the WT strain (Fig. S5B–D). The results of pathogenicity assays using *C. oleifera* leaves revealed that the *CaSUN1*^Δ*1,3AIM*/*LIR*^*-C* /Δ*Casun1* mutant strain exhibited attenuated virulence and that lesions were less severe compared to those of the WT strain (Fig. S5E). These findings suggested that the first and third AIM/LIR motifs of CaSun1 are essential for its biological function and intracellular localization in *C. camelliae*.

## Discussion

Emerging evidence suggests that mitophagy plays a vital role in the virulence of various plant pathogenic fungi, including *M. oryzae*, *U. virens*, *C. higginsianum*, and *Beauveria bassiana* [7, 8, 13, 27, 39]. However, the significance of mitophagy in pathogenic fungi remains to be elucidated. Atg8, also known as LC3, plays a crucial role in the autophagic processes of eukaryotic cells [14, 32, 35]. CaSun1, a protein interactor of CaAtg8, was identified in this study by IP-MS. The findings demonstrate that CaSun1 regulates fungal growth, conidial morphology, and pathogenicity in *C. camelliae*. Specifically, CaSun1 interacts with CaAtg8 via AIM/LIR motifs and facilitates the recruitment of autophagosomes to enhance mitophagy, thereby regulating the virulence of *C. camelliae*.

*Colletotrichum camelliae* is a fungal pathogen pertinent to the *Colletotrichum* genus, which is notorious for causing anthracnose in various plants, particularly the tea oil tree [40–42]. Anthracnose poses a significant threat to agricultural stability and crop yield, especially in regions where tea oil is a major cash crop [19, 24, 43]. The process of infection with *C. camelliae* initiates with conidial germination, followed by the dispersal of asexual spores by wind or rain, which must then encounter a suitable host to establish infection. Following initial attachment to the surface of the host plant, the conidia germinate and form appressoria, which are specialized structures that facilitate the penetration of host cuticles. *Colletotrichum camelliae* subsequently establishes an invasive growth phase characterized by the production of intracellular fungal structures and invasive hyphae, which proliferate within the host tissue. The typical fused lesions are visible on the leaves of tea oil trees after 7 days [22, 44, 45]. Understanding the mechanism underlying the pathogenicity of *C. camelliae* is critical for the development of effective management strategies aimed at mitigating its destructive effects on plant health and agricultural productivity.

**Figure 7:**
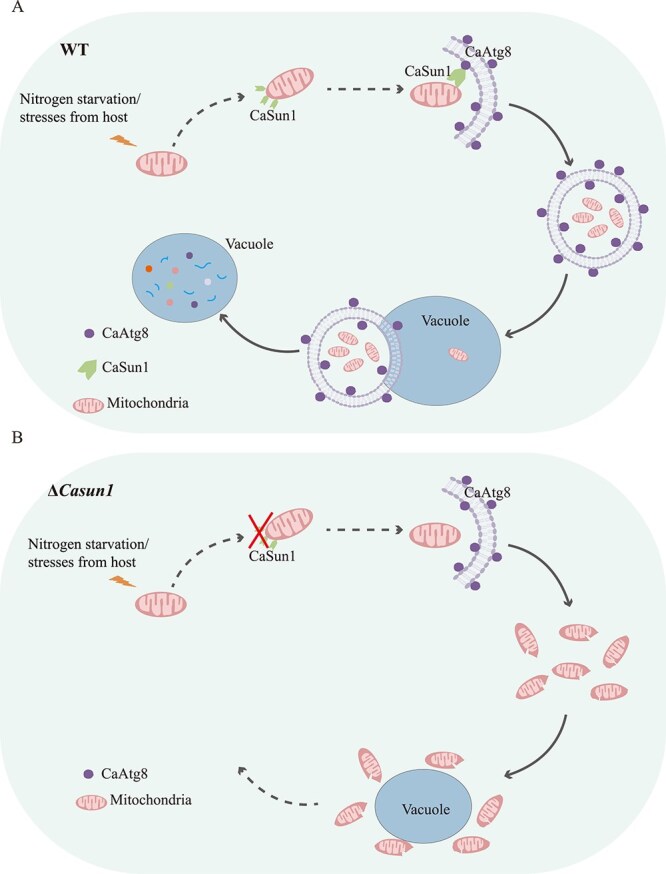
**Proposed model of CaSun1-CaAtg8 function in nitrogen starvation-induced mitophagy.** (**A**) Nitrogen starvation induces mitochondrial rupture and dysfunctional mitochondria, urging that CaSun1 recruits CaAtg8 to initiate the process of mitophagy. The autophagosomes encapsulating the mitochondria then fuse with vacuoles before degradation. (**B**) The Δ*Casun1* mutant cannot efficiently remove dysfunctional mitochondrial by mitophagy in time, leading to damaged mitochondrial accumulation and abnormal mycelial physiology.

Mitophagy is a critical cellular process that ensures the maintenance of mitochondrial quality and function [16, 46]. This process is tightly regulated by various signaling pathways and is influenced by cellular stress, nutrient availability, and developmental cues [47]. The autophagy-related proteins (ATGs) and mitochondrial proteins of *M. oryzae*, including MoAtg24, MoDnm1, MoFis1, MoMdv1, MoAuh1, and MoAti1, constitute the core machinery of mitophagy [8–11]. These components collaborate to identify damaged mitochondria, recruit the autophagic machinery, and facilitate the degradation of organelles [1, 2]. It has been reported that ScUth1, a member of the SUN protein family in yeast, plays a role in rapamycin response under respiratory conditions with lactate as the sole carbon source, suggesting its involvement in mitophagy [30]. Additionally, Sun1p has been implicated in the selective recognition of dysfunctional mitochondria, facilitating their engulfment by the autophagic machinery [31]. However, another study reported that ScUth1 is dispensable for mitophagy, thus rendering its role controversial [29]. The present study identified that CaSun1, a member of the SUN family, is involved in mitophagy in *C. camelliae*. Sequence analysis revealed that *C. camelliae* has only one SUN family protein, unlike *S. cerevisiae*, which contains four SUN family proteins (Fig. S1). Further evidence supported the hypothesis that CaSun1 recruits CaAtg8 to the mitochondria to initiate mitophagy in *C. camelliae* (Fig. 5). First, the results of IP-MS identified that CaSun1 is a potential protein interactor of CaAtg8, and is essential for vegetative growth, conidiation, and virulence (Fig. 2). Second, the findings revealed that CaSun1 interacts with CaAtg8 both *in vitro* and *in vivo*. CaSun1 was also found to localize to the mitochondria and was specifically involved in mitophagy, but not in autophagy under conditions of nitrogen starvation (Fig. 5). Third, it was observed that a point mutant in CaSun1 disrupted the interaction between CaSun1 and CaAtg8 (Fig. 6 and Fig. S5A), resulting in defective of mycelia growth, conidiation, and pathogenicity. Altogether, the findings revealed that CaSun1 regulates mitophagy by recruiting CaAtg8 to mitochondria and may represent the first mitophagy-related gene identified in *C. camelliae*.

In conclusion, this study provides a working model of the association between CaSun1 and CaAtg8 in *C. camelliae*. During nitrogen starvation, CaSun1, a member of the SUN protein family, interacts with CaAtg8 via 1,3 AIM/LIR motifs and facilitates the recruitment of autophagosomes, resulting in the selective degradation of dysfunctional mitochondria by mitophagy (Fig. 7). It was additionally observed that CaSun1 specifically participates in mitophagy to regulate asexual development, conidiation, and virulence. Our findings largely confirm the functions of a SUN family protein in mitophagy, enhance our understanding of the mechanisms underlying the role of mitophagy in fungal pathogenesis, and provide novel therapeutic strategies for the management of anthracnose-causing fungi.

## Material and methods

### Fungal strains and culture conditions

Professor Junbin Huang of Huazhong Agriculture University generously contributed to the *C. camelliae* wild-type (WT) strain (Hubei Province, China). All employed *C. camelliae* strains were cultured at 25°C on potato dextrose agar (PDA) (potato 200 g/l, glucose 20 g/l, and agar 20 g/l) plates. Fungal mycelia were grown in liquid PDB (potato 200 g/l, glucose 20 g/l) at 25°C with shaking for 40 h and then the mycelia were harvested for genomic DNA, RNA, or protein extraction.

### Plasmid constructs and fungal transformants

Mutants of *CaSUN1* were generated by amplifying the 5’ UTR and 3’ UTR of *CaSUN1* from the WT strain genomic DNA and integrating them into the flanking regions of a *hygromycin* resistance gene cassette within the pGKO plasmids. The resultant plasmid was subsequently introduced into the WT strain by the *Agrobacterium tumefaciens-*mediated transformation (ATMT) method [32]. Positive transformations were validated with polymerase chain reaction (PCR), quantitative real-time PCR (qRT-PCR), and Southern blot assays. An RFP tag was ligated to the C-terminus of CaSun1 to generate CaSun1-RFP. The resulting plasmid was then transformed into the WT strain, resulting in RFP integration at the CaSun1 genomic locus.

For complementation analysis, the full-length genomic copy, including the *CaSUN1* promoter, was cloned and inserted into p3300neo-GFP and then transformed into ∆*Casun1* by ATMT. Similarly, the *CaSUN1^∆1,3AIM^*-C complementation constructs were generated and transformed into the ∆*Casun1* strain to obtain the resulting *CaSUN1^∆1,3AIM^*-C/∆*Casun1* strain. PCR and qRT-PCR performed verification of positive transformants (Table S1).

### Assayment for vegetative growth, conidiation, and appressorium formation

To assess vegetative development, 3-mm^2^ mycelial plugs from fresh cultured strains were placed on PDA agar plates followed by a 2-day incubation before exposure to continuous light for 5 days. To evaluate stress responses, *C. camelliae* strains were inoculated on PDA medium as well as on PDA medium containing 0.4 M NaCl, 0.04% SDS (Sodium Dodecylsulphate), 200 μg/ml CR (Congo red), and 200 μg/ml CFW (Calcofluor White), and placed at 25°C for 7 days, respectively. The previously mentioned formula was employed to determine the inhibition rate [33]. For conidiation, an inoculating loop was used to scrape the surface of the colonies, and the suspension underwent filtration through three layers of lens paper to exclude mycelial debris. Under a microscope, the conidia were counted using a hemocytometer after being cleaned three times with deionized water. Conidial suspensions were maintained in an incubator at 25°C without light on glass slides (hydrophobic films) to facilitate the production of appressorium. The germination and appressorium production rates were examined under a microscope after 24 h. Every experiment was carried out using three biological duplicates in triplicate.

### Inoculation assay

Mycelia blocks (3 mm^2^) from fresh cultured strains were grown on PDB agar for 2 days. The conidial suspensions (1 × 10^6^ spores/ml) were collected and inoculated onto leaves of *C. oleifera* ‘Changlin 40’, which were then placed in an incubator for 7 days at 25°C with a 14/10-h light/dark cycle. After incubation, images of the diseased leaves were captured, and the relative lesion area was quantified using Image J.

### Generation of the GFP-CaAtg8 strain and LC–MS/MS analysis

To generate the fluorescent localization of GFP-CaAtg8, 1-kb fragment of the 3’ UTR sequences of *CaATG8* underwent amplification from the WT strain *HBMC-171* genomic DNA and ligated to pGKO. Subsequently, the full-length genomic copy composed of 1.2 kb of the promotor, eGFP, and the coding region of *CaATG8* were inserted into the previous construct to produce the GFP-CaAtg8 plasmid. The correct plasmid was introduced into the WT strain by ATMT. Positive transformants were achieved by GFP fluorescence or western blot analysis.

To identify potential interactors of CaAtg8, total proteins from GFP-CaAtg8-expressing cells were extracted with lysis buffer (50 mM Tris–HCl pH 7.4, 1 mM EDTA, 100 mM NaCl, 1% Triton 100, and 1 × proteinase inhibitor) and centrifuged at 12 000 rpm for 10 min at 4°C. The supernatant containing GFP-CaAtg8 was incubated with 30 μl of GFP-Trap magnetic beads according to the manufacturer’s instructions. Following incubation, beads were thrice rinsed in dilution buffer, and CaAtg8 was eluted. The eluted proteins were then sent to Shanghai Luming Biological Technology Co., Ltd. (Shanghai, China) for digestion, concentration, desalting, and analysis using a Thermo Scientific LTQ Velos mass spectrometer.

### Protein interaction analysis *in vivo* and *in vitro*

The bait plasmid pGADT7-CaSun1 for the yeast two-hybrid experiment was generated by cloning the full-length CaSun1 cDNA into pGBKT7 using primers Sun1-ADF/ADR. Similarly, CaAtg8 was cloned into pGBKT7 using primers Atg8-BDF/BDR to get the prey plasmid pGBKT7-CaAtg8. After confirming both plasmids via sequencing, these were cotransformed into the yeast strain AH109 using the lithium acetate transformation method. Yeast cells were cultivated on SD media lacking both tryptophan and leucine, and interaction was measured by SD-Trp/Leu/His/Ade medium growth. pGBKT7-p53 and pGADT7-T pairs were utilized as a positive control, and pGBKT7-lam and pGADT7-T were used a negative control.

To further investigate the interaction between CaSun1 and CaAtg8, a bimolecular fluorescence complementation (BiFC) assay was conducted. The CaSun1-YFPN and YFPC-CaAtg8 fusion proteins were constructed for the BiFC assay as previously reported [34] and cotransformed into the *C. camelliae* strains. As a negative control, combinations of YFPN and YFPC were used in this section.

To confirm the interaction between GFP-CaAtg8 and CaSun1-Flag *in vivo*, a co-immunoprecipitation (Co-IP) assay was performed. Strains coexpressing GFP-CaAtg8/CaSun1-Flag were cultured in a liquid PDB medium for 40 h. Then the total proteins were extracted and treated with 30 μl of GFP-Trap magnetic beads according to the manufacturer’s instructions. The beads were then subjected to three washes with a buffer of 50 mM Tris–HCl (PH 7.5), 150 mM NaCl, and 5 mM EDTA buffer to eliminated nonassociated proteins. Sodium dodecyl sulfate-polyacrylamide gel electrophoresis (SDS-PAGE) separated the proteins bound to the beads and the input protein samples, followed by transfer to a membrane and analysis using western blot using anti-GFP and anti-Flag antibodies for GFP-CaAtg8 and CaSun1-Flag detection, respectively.

### Autophagy and mitophagy analysis

For autophagy assays, the *GFP-CaATG8* strain was cultivated in PDB for 40 h, followed by a wash and a nitrogen starvation experiment in which the cells were transferred to an SD-N medium for 6 or 12 h to trigger autophagy. Following 6 h of induction, GFP-CaAtg8 cleavage was analyzed biochemically, and mycelia were inspected under a microscope. Immunoblotting for GFP-CaAtg8 cleavage was conducted with anti-GFP and anti-GAPDH antibodies.

For mitophagy assays, the *CaMTS-GFP* strain was cultured in PDB for 40 h, prior to transfer to SD-N medium for 6 or 12 h to induce mitophagy. Total proteins were electrophoresed by 10% SDS-PAGE, followed by western blotting with an anti-Porin antibody. For vacuolar staining, mycelia were stained for 30 min with 20 μM CellTracker™ Blue CMAC Dye (7-amino-4-Chloromethylcoumarin, Molecular Probes, C2110) at room temperature, rinsed in deionized water, and observed using a Zeiss LSM 880 microscopy (Zeiss, Oberkochen, Germany).

## Supplementary Material

Web_Material_uhaf121

## Data Availability

The data presented in this study are available in the article and in its online supplementary material.
