# Supplementary material for: CaSun1, a SUN family protein, governs the pathogenicity of Colletotrichum camelliae by recruiting CaAtg8 to promote mitophagy
Source: Hortic Res. 2025 May 2;12(7):uhaf121. doi: 10.1093/hr/uhaf121 (PMC12136678; doi:10.1093/hr/uhaf121)
Supplement: Web_Material_uhaf121 [file web_material_uhaf121.zip › Figure S.docx]

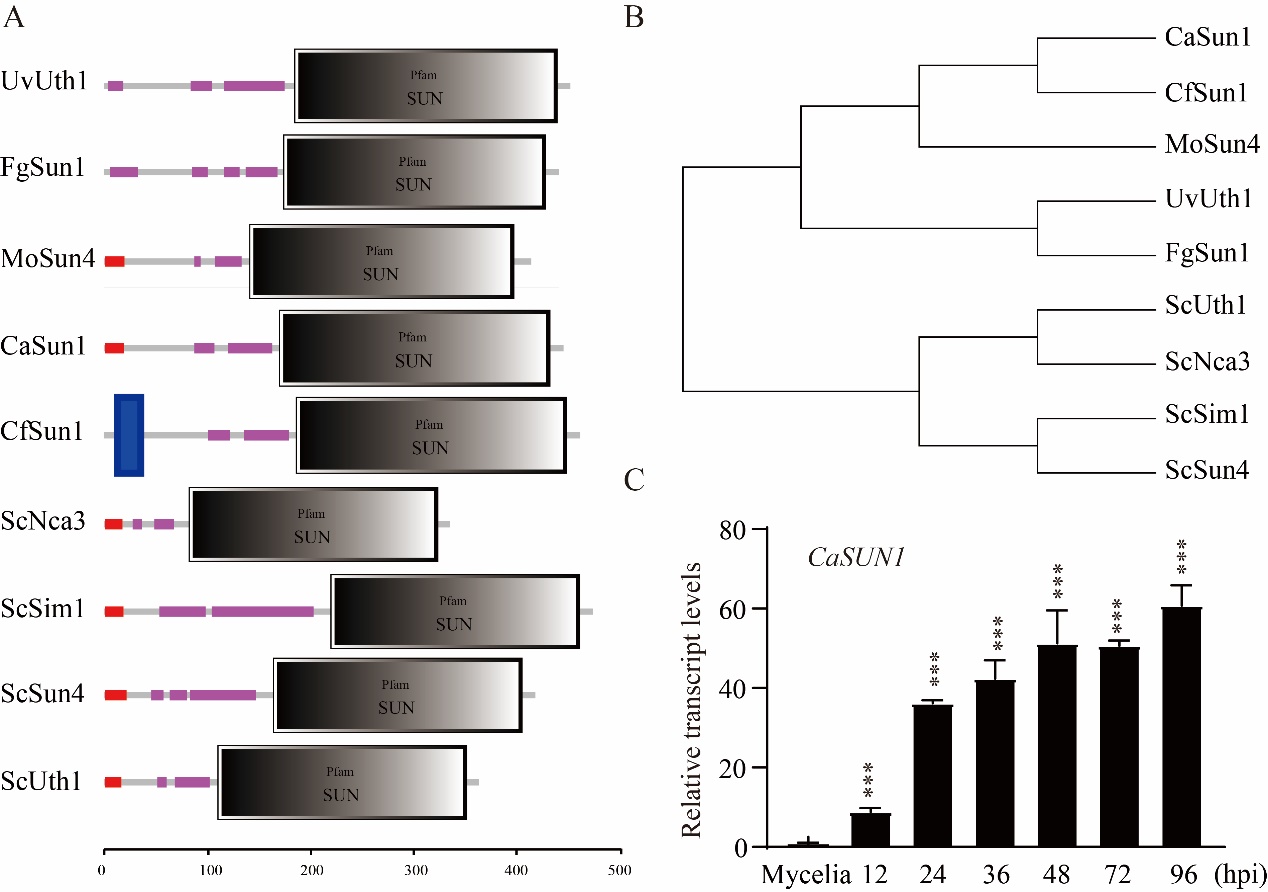


**Fig. S1 Identification of *CaSUN1* in *C. camelliae*. (A)** The SUN family proteins possess a conserved SUN domain. **(B)** A phylogenetic tree of SUN family proteins from various fungi was constructed using MEGA 10.0 with the neighbor-joining algorithm. **(C)** The relative expression levels of *CaSUN1* are upregulated during the infection stage, with *β-ACTIN* gene as an internal control. Data are presented as the mean ± SD from three biological replicates and analyzed using Student’s T-test. Statistical significance is indicated as "***" (P < 0.001).


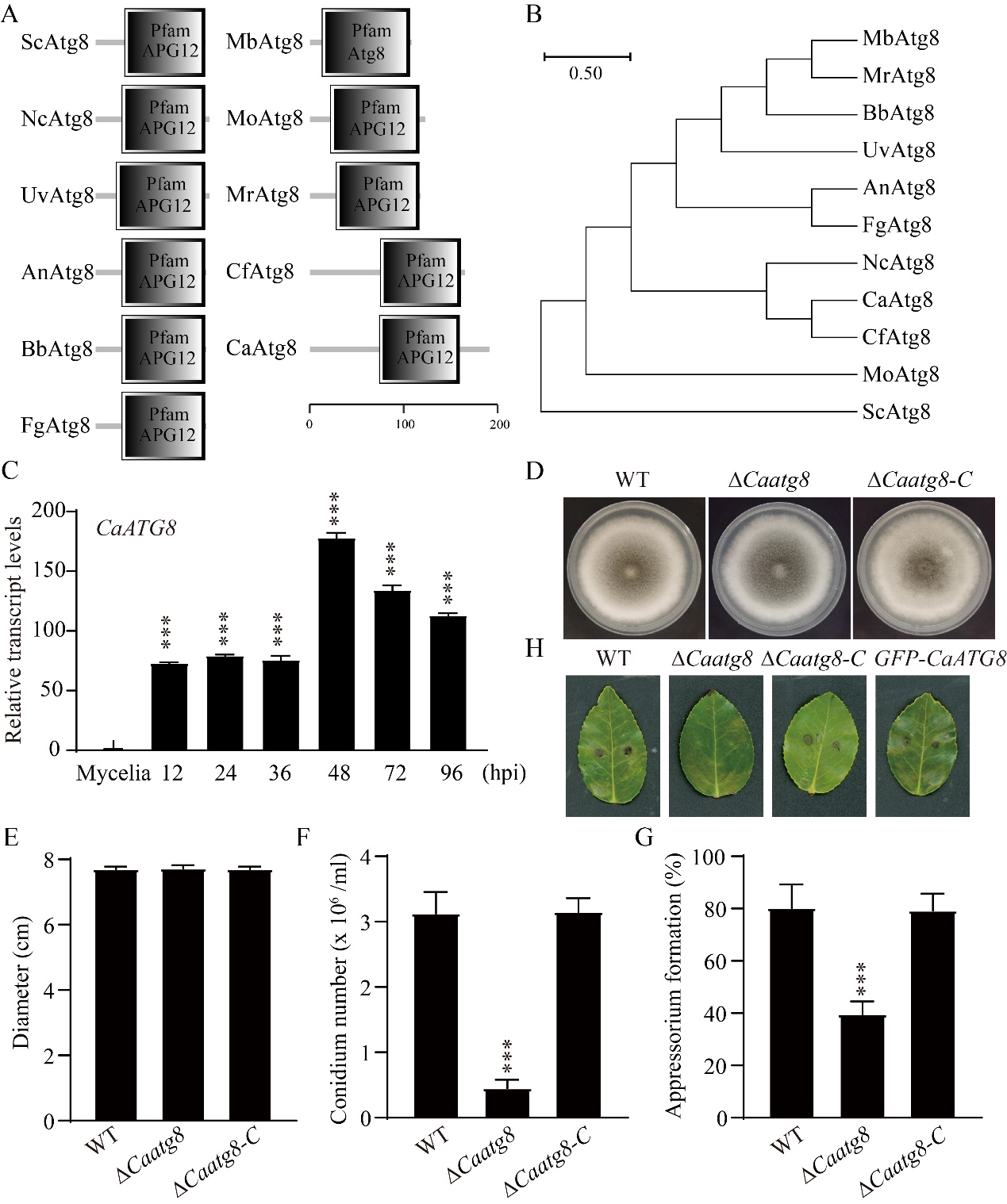


**Fig. S2 Identification of *CaATG8* and Δ*Caatg8*** **mutant is defective in conidiation and pathogenicity in *C. camelliae*. (A)** Functional domain analysis of Atg8 homologs. **(B)** Phylogenetic tree of Atg8 homologs from several organisms were constructed using MEGA 10.0 with the neighbor-joining algorithm. **(C, D)** The colony morphology of the wild type, Δ*Caatg8*, and the complementation strain in PDA medium at 25°C for 7 days. **(E)** Conidiation was impaired in Δ*Caatg8* strain. Conidia were collected and counted on PDB medium cultures of the wild type, Δ*Caatg8*, and the complementation strain. The values were derived from three biological replicates and repeated three times. **(F)** The percentage of conidia forming appressorium in each strain. **(G)** Disease symptoms were observed after inoculation with conidial suspensions (1×10^6^ spores/mL) from WT, Δ*Caatg8*, and Δ*Caatg8*-C strains. Mean and SD were calculated from three biological replicates. Significant at ***p < 0.001.


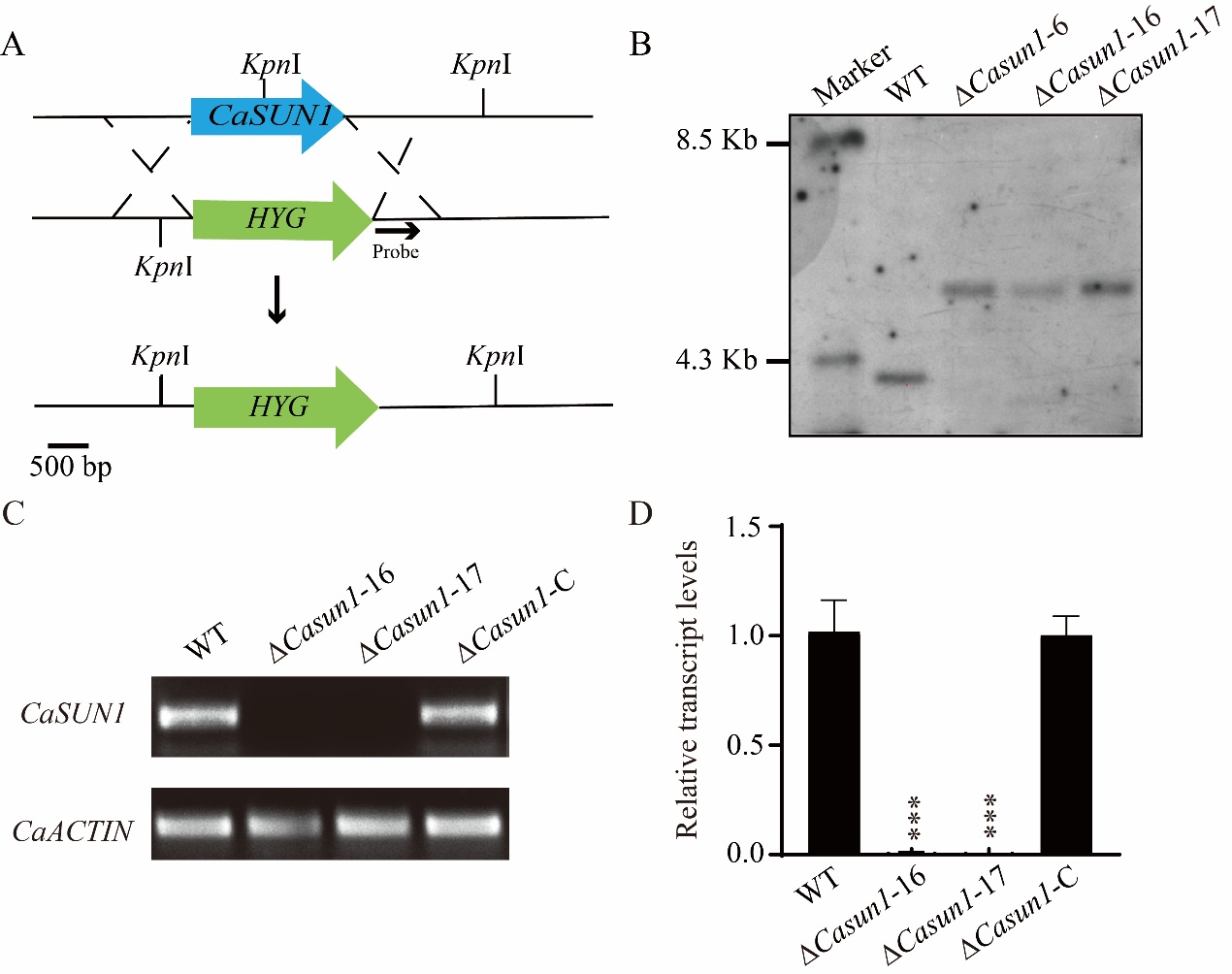


**Fig. S3 Construction strategies for *CaSUN1* knockout vector and Southern blot assay. (A)** Schematic representation of the gene deletion strategy. **(B)** Southern blot analysis of wild-type and Δ*Casun1* mutants. Genomic DNA isolated from the WT and Δ*Casun1* mutants were digested by *Kpn* I, and then hybridized with the probe as shown in the figure S3A. **(C)** RT-PCR analysis of Δ*Casun1* mutant strains. **(D)** qRT-PCR analysis of *CaSUN1* expression in the WT, Δ*Casun1* mutants, and complemented strain. Data are from three biological replicates. Asterisks indicate significant differences at a p-value < 0.001.


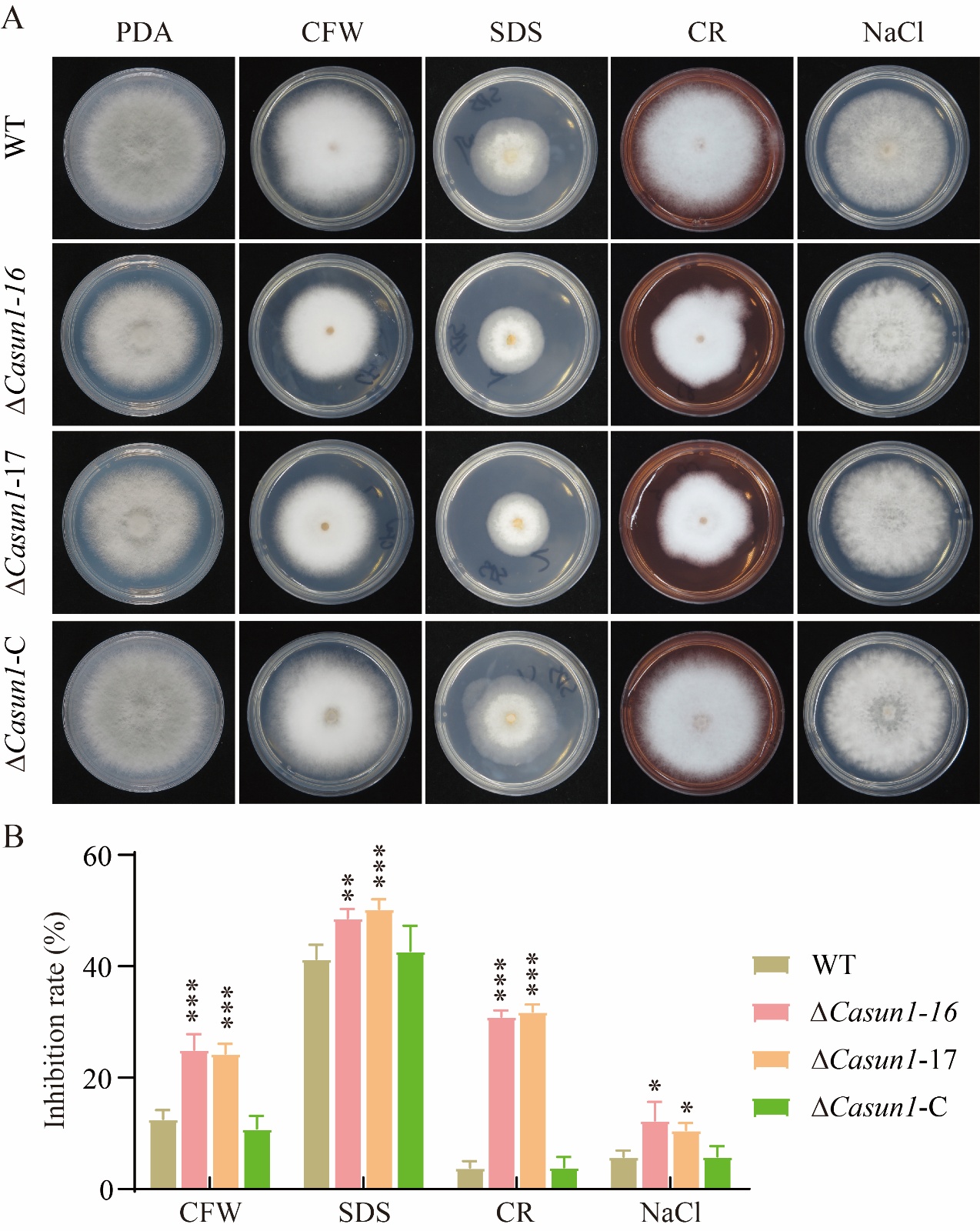


**Fig. S4 Sensitivity test of the wild-type,** **Δ*Casun1*, Δ*Casun1*-C strains to various stresses. (A)** The representative strains were cultured on PDA and PDA containing various stressors: 0.4 M NaCl, 0.04% SDS, 200 μg/mL CR, and 200 μg/mL CFW at 25°C for 7 days. **(B)** Statistical analysis of the inhibition rates of test strains under different stress conditions, colony diameters were measured and analyzed. There were consistent findings from three tests (p-value < 0.001 level).


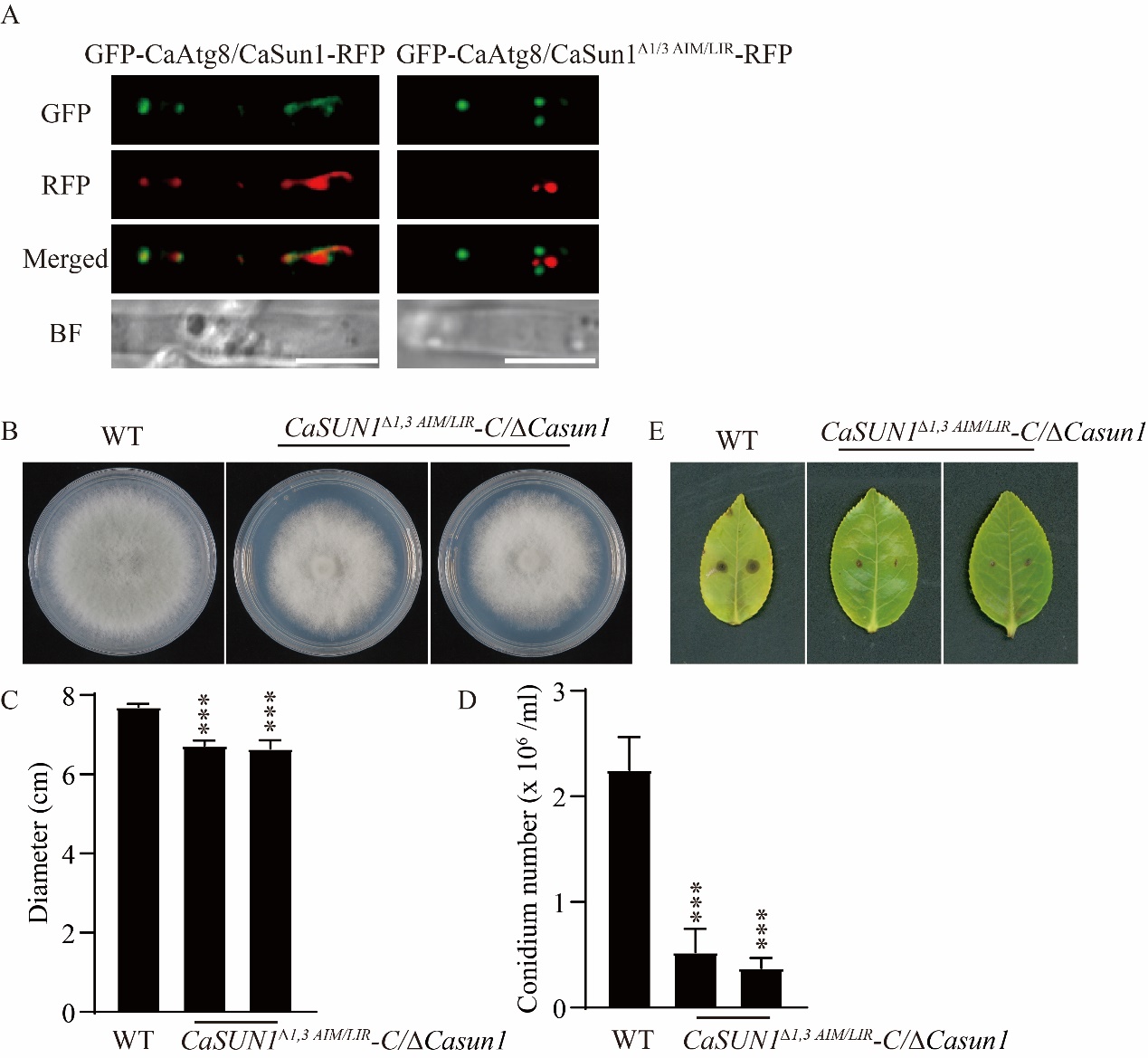


**Fig. S4 AIM motifs is important for CaSun1. (A)** AIM/LIR motifs are required for the co-localization of CaSun1 and CaAtg8. CaSun1-RFP co-localized with GFP-CaAtg8 on autophagosomes (dotted fluorescence) under nitrogen starvation condition. Mutations in the first and third AIM/LIR motifs disrupted the co-localization of CaSun1-RFP and GFP-CaAtg8. **(B, C)** Mutations in the first and third AIM/LIR motifs of *CaSUN1* decreased the colony diameter in comparison with that of the wild-type strain. **(D)** Conidiation was impaired in *CaSUN1*^Δ^*^1,3AIM/LIR^-C*/Δ*Casun1* strain. **(E)** Disease symptoms on leaves were observed after inoculation with conidial suspensions (1×10^6^ spores/mL) from WT, Δ*Casun1*, and Δ*Casun1*-C strains in a humidified chamber.
