# Supplementary material for: CaSun1, a SUN family protein, governs the pathogenicity of Colletotrichum camelliae by recruiting CaAtg8 to promote mitophagy
Source: Hortic Res. 2025 May 2;12(7):uhaf121. doi: 10.1093/hr/uhaf121 (PMC12136678; doi:10.1093/hr/uhaf121)
Supplement: Web_Material_uhaf121 [file web_material_uhaf121.zip › Table S1.docx]

Table S1 Primers used in this study.

| **Primer** | **Sequence (5’-3’)** | **Application** |
| --- | --- | --- |
| CaSun1-5F  CaSun1-5R  CaSun1-3F  CaSun1-3R  CaSun1-TF  Hyg-5R  CaSun1-cf  CaSun1-cr  CaSun1-pRFPF  CaSun1-pRFPR  CaSun1-CDSF  CaSun1-RFPR  CaSun1RT-F  CaSun1RT-R  CaACTIN-F  CaACTIN-R  CaSun1-probeF  CaSun1-probeR  CaSun1-ADF  CaSun1-AD1R  CaSun1-AD1F  CaSun1-AD2R  CaSun1-AD2F  CaSun1-AD3R  CaSun1-AD3F  CaSun1-AD4R  CaSun1-AD4F  CaSun1-AD5R  CaSun1-AD5F  CaSun1-ADR  CaAtg8-BDF  CaAtg8-BDF  Patg8-F  Patg8-R  Gatg8-F  Gatg8-R  Atg8-F  Atg8-R  CaSun1-FlagF  CaSun1-FlagR  CaSun1-YNF  CaSun1-YNR  YC-CaAtg8F  YC-CaAtg8R  AIM-CF  AIM1-CR  AIM-1CF  AIM-3CR  AIM-3CF  AIM-R | attattatggagaaactcgagTGGCTGGACCTGGGAAAATC ctagtggatcccccgggtaccTTTCGTGCGATCAACTCCCA  ccggaaccagtcgacctgcagGGCGAGGTTTTTGGGCATTT acgacggccagtgccaagcttAACGAGCAGCAGTCTTACCC  AACGAGGAAAACAGAGCCGT  ACCTCCACTAGCTCCAGCCAAG  tatggagaaactcgagaattcAACGAGGAAAACAGAGCCGT  ctagtggatcccccgggtaccTCTTGCTTTCCAGTGAGGT  tatggagaaactcgagaattcGCATTCTTGCATCGAGCCTG  GACGATGGACCTCATTTCGTGCGATCAACTCCCA  GCACGAAATGAGGTCCATCGTCAGCCTCACCAT  ctcgcccttgctcacggtaccGTAGAAGCGGATGATGGCAGTG CCCTGTTGGCTACCAGAAGA  TGAGGTCGTTCTGGACGTAG  GGTAACCAAATCGGTGCTGCTTTC  CCCGCATCTGGTAGACAAGA  GGCGAGGTTTTTGGGCATTT  AACGAGCAGCAGTCTTACCC  gccatggaggccagtgaattcATGAGGTCCATCGTCAGCCTC  TCCagcgacaacggcCAGGCCGTCGTTGATGCC  CTGgccgttgtcgctGGAGAGACCACCCCGTCCT  agcggcacctgcGTCGGACGGGAACGTGGA  TCCGACgcaggtgccgctCCCGTCGACTACCTCGGC  TCTCTGagcgccggttgcGCCGTTGGTGCCGAGGTA  gcaaccggcgctCAGAGAACCCCCAACTACAGCA  TTagcgggagctgcGTTACCGGCAGCGAGGGG  TAACgcagctcccgctAACATTGGTGTTGGCAAGAGC  TTGAAcgcggagagtgcGGTGTTGCCGTCGGCGCT  CgcactctccgcgTTCAACAATGCCCCGACCT  cagctcgagctcgatggatccTCAGTAGAAGCGGATGATGGC  atggccatggaggccgaattcATGCGATCCAAGTTCAAGGACG  ccgctgcaggtcgacggatccCTACTCGCGGGGGAACCC  attattatggagaaactcgagCATGATGCGGCCGGATAG  tgctcaccatGATGAAGGTGTTGGTAACGGGC  caccttcatcATGGTGAGCAAGGGCGAGG  tggatcgcatCTTGTACAGCTCGTCCATGCC  gctgtacaagATGCGATCCAAGTTCAAGGACG  ctagtggatcccccgggtaccCTACTCGCGGGGGAACCC  aatcttcaaacccgggctgcaggaattcAACGAGGAAAACAGAGCCGT  atcgataagcttgatatcgaattcCTACTCGCGGGGGAACCC  aaccgtcaaaatggtcggatccATGAGGTCCATCGTCAGCCTC  tggagcgcccggggatggatccTCAGTAGAAGCGGATGATGGC  caccatcacgccatggtcgacATGCGATCCAAGTTCAAGGACG  ttctgcttgtccatcactagtCTACTCGCGGGGGAACCC  tatggagaaactcgagaattcGCATTCTTGCATCGAGCCTG  TCCagcgacaacggcCAGGCCGTCGTTGATGCC  CTGgccgttgtcgctGGAGAGACCACCCCGTCCT  TCTCTGagcgccggttgcGCCGTTGGTGCCGAGGTA  gcaaccggcgctCAGAGAACCCCCAACTACAGCA  ctcgcccttgctcacggtaccGTAGAAGCGGATGATGGCAGTG | Amplifying 5’ flank sequence of *CaSUN1* for gene deletion  Amplifying 3’ flank sequence of *CaSUN1* for gene deletion  Transformants screening  Construction of complementation vector  Construction of fluorescent localization vector  Analysis the expression level of *CaSUN1* gene of *Colletotrichum camelliae*  Analysis the expression level of *CaACTIN* gene of *Colletotrichum camelliae*  Amplification of probe for Southern blot assay  Construction of AD-CaSun1^Δ1AIM/LIR^  Construction of AD-CaSun1^Δ2AIM/LIR^  Construction of AD-CaSun1^Δ3AIM/LIR^  Construction of AD-CaSun1^Δ4AIM/LIR^  Construction of AD-CaSun1^Δ5AIM/LIR^  Construction of BD-CaAtg8  Construction of fluorescent localization vector of GFP-CaAtg8  Construction of CaSun1-Flag  Construction of CaSun1-YN  Construction of YC-CaAtg8  Construction of complementation vector of CaSun1^1AIM^-C/Δ*Casun1*  Construction of complementation vector of CaSun1^3AIM^-C/Δ*Casun1* |

Table S2 Proteins identified by IP-MS analysis.

| Accession | Description | Score Sequest HT | Coverage | # Peptides | # Unique Peptides | MW [kDa] | calc. pI |
| --- | --- | --- | --- | --- | --- | --- | --- |
| KAH0432570.1 | beta-glucosidase | 89.9215734 | 29.326923 | 18 | 18 | 45.57 | 5.76 |
| KAH0432843.1 | hypothetical protein CcaCcLH18_06127 | 67.2905153 | 40.38055 | 14 | 14 | 51.81 | 8.23 |
| KAH0421422.1 | mRNA cleavage factor complex component pcf11 | 57.592592 | 33.892216 | 11 | 11 | 75.58 | 9.42 |
| KAH0424354.1 | peptidase family M20/M25/M40 protein | 56.86414433 | 19.625 | 13 | 13 | 89.05 | 9.98 |
| KAH0431708.1 | flap structure-specific endonuclease | 53.55777824 | 20.365854 | 14 | 14 | 90.48 | 8.59 |
| KAH0420231.1 | glycosyl hydrolase family 38 domain-containing protein | 49.80007923 | 24.319419 | 13 | 13 | 123.92 | 5.54 |
| KAH0421810.1 | glycolipid 2-alpha-mannosyltransferase | 44.20970488 | 10.416667 | 6 | 6 | 58.47 | 6.4 |
| KAH0429929.1 | 5-methyltetrahydropteroyltriglutamate- homocysteine methyltransferase | 37.53871822 | 27.590512 | 15 | 15 | 86.49 | 6.86 |
| KAH0434294.1 | eukaryotic translation initiation factor | 33.71503222 | 5.8441558 | 1 | 1 | 17.7 | 5.05 |
| KAH0424122.1 | hypothetical protein CcaCcLH18_11767 | 31.85541105 | 30.894309 | 12 | 12 | 80.76 | 5.84 |
| KAH0423330.1 | DSBA-like thioredoxin domain-containing protein | 25.37130928 | 18.770227 | 5 | 5 | 31.47 | 9.98 |
| KAH0424782.1 | hypothetical protein CcaCcLH18_11338 | 24.93898535 | 4.989605 | 1 | 1 | 51.29 | 10.03 |
| KAH0434413.1 | fyve zinc finger protein | 23.3562579 | 19.864574 | 6 | 6 | 62.96 | 5.67 |
| KAH0433615.1 | hypothetical protein CcaCcLH18_05787 | 22.64025486 | 17.725753 | 13 | 13 | 99.98 | 5.74 |
| KAH0425516.1 | hypothetical protein CcaCcLH18_10899 | 14.21686747 | 10.008057 | 6 | 6 | 49.23 | 5.27 |
| KAH0436990.1 | alpha-mannosidase | 11.45149601 | 6.4461408 | 8 | 8 | 129.93 | 6.27 |
| KAH0445053.1 | alkaline phosphatase family protein | 11.45149601 | 6.4461408 | 8 | 8 | 127.16 | 4.95 |
| KAH0424784.1 | Dor1-like family protein | 6.660464287 | 4.0268456 | 4 | 4 | 48.41 | 5.46 |
| KAH0429297.1 | hypothetical protein CcaCcLH18_08509 | 6.255628109 | 17.866667 | 6 | 6 | 42.16 | 4.62 |
| KAH0433614.1 | mitogen-activated protein kinase sty1 | 6.255628109 | 17.866667 | 6 | 6 | 41.42 | 5.64 |
| KAH0421207.1 | hypothetical protein CcaCcLH18_13561 | 6.09916842 | 7.3333333 | 4 | 4 | 60.15 | 9.97 |
| KAH0422437.1 | hypothetical protein CcaCcLH18_12812 | 5.994051695 | 9.7222222 | 2 | 2 | 25.18 | 9.04 |
| KAH0431243.1 | hypothetical protein CcaCcLH18_07268 | 5.897742748 | 6.0165975 | 3 | 3 | 53.14 | 7.16 |
| KAH0443807.1 | protein kinase c | 5.700214863 | 4.0833333 | 5 | 5 | 129.79 | 7.77 |
| KAH0431952.1 | XPG domain-containing protein | 4.213637471 | 8.2294264 | 1 | 1 | 44.69 | 8.19 |
| KAH0434636.1 | serine threonine-protein kinase psk1 | 4.209704876 | 10.416667 | 6 | 6 | 58.01 | 7.69 |
| KAH0435674.1 | sphingomyelinase | 3.761310935 | 6.1746988 | 3 | 3 | 71.47 | 5.34 |
| KAH0423857.1 | hypothetical protein CcaCcLH18_11913 | 3.71503222 | 8.8888889 | 1 | 1 | 13.53 | 6.5 |
| KAH0440436.1 | hypothetical protein CcaCcLH18_02460 | 3.660837293 | 3.8302277 | 4 | 4 | 106.71 | 5.41 |
| KAH0443497.1 | hypothetical protein CcaCcLH18_00810 | 3.606953502 | 5.6497175 | 4 | 4 | 81.04 | 9.9 |
| KAH0441975.1 | hypothetical protein CcaCcLH18_01835 | 2.457074642 | 1.9455253 | 1 | 1 | 87.58 | 8.88 |
| KAH0439402.1 | aldose 1-epimerase | 1.975669742 | 4.1666667 | 1 | 1 | 36.52 | 4.92 |
